# Preliminary Evaluation of Radiomics in Contrast-Enhanced Mammography for Prognostic Prediction of Breast Cancer

**DOI:** 10.3390/cancers17121926

**Published:** 2025-06-10

**Authors:** Luca Nicosia, Luciano Mariano, Aurora Gaeta, Sara Raimondi, Filippo Pesapane, Giovanni Corso, Paolo De Marco, Daniela Origgi, Claudia Sangalli, Nadia Bianco, Serena Carriero, Sonia Santicchia, Enrico Cassano

**Affiliations:** 1 Division of Breast Radiology, Department of Medical Imaging and Radiation Sciences, IEO European Institute of Oncology, IRCCS, Via Ripamonti 435, 20141 Milano, Italy; luciano.mariano@ieo.it (L.M.); filippo.pesapane@ieo.it (F.P.); enrico.cassano@ieo.it (E.C.); 2 Department of Experimental Oncology, IEO European Institute of Oncology IRCCS, 20141 Milano, Italy; aurora.gaeta@ieo.it (A.G.); sara.raimondi@ieo.it (S.R.); 3 Department of Statistics and Quantitative Methods, University of Milano-Bicocca, 20126 Milano, Italy; 4 Division of Breast Surgery, IEO European Institute of Oncology IRCCS, 20141 Milano, Italy; giovanni.corso@ieo.it; 5 Department of Oncology and Hemato-Oncology, University of Milan, 20122 Milano, Italy; 6 Medical Physics Unit, IEO European Institute of Oncology IRCCS, 20141 Milano, Italy; paolo.demarco@ieo.it (P.D.M.); daniela.origgi@ieo.it (D.O.); 7 Clinical Trial Office, IEO European Institute of Oncology IRCCS, 20141 Milano, Italy; claudia.sangalli@ieo.it; 8 Division of Medical Senology, IEO European Institute of Oncology IRCCS, 20141 Milano, Italy; nadia.bianco@ieo.it; 9 Radiology Department, Fondazione IRCCS Cà Granda Ospedale Maggiore Policlinico, Via Francesco Sforza 35, 20122 Milano, Italy; serena.carriero@policlinico.mi.it (S.C.); sonia.santicchia@policlinico.mi.it (S.S.)

**Keywords:** mammography, radiomics, disease-free survival, survival rate, breast neoplasm

## Abstract

Background: Radiomics is changing clinical practice by providing quantitative information from images to improve diagnosis, prognosis, and treatment planning. This study aims to investigate a radiomics model developed from contrast-enhanced mammography (CEM) images to predict disease-free survival (DFS) and overall survival (OS) in breast cancer (BC) patients. Methods: From January 2013 to December 2015, all consecutive BC patients who underwent CEM before biopsy at a referral center were enrolled. Clinical data included histological results, receptor profiles, and follow-up (DFS and OS). A region of interest (ROI) of the enhancing lesion was selected from recombined CEM images by experienced radiologists, and radiomic features were extracted. A Cox-LASSO model assigned coefficients to the features, generating patient radiomic scores (RSs), which were dichotomized for graphical representation. Model performance was assessed using the C index. Results: The study included 126 BC patients with predominantly “mass”-type lesions (95%) and a median follow-up of 6.88 years (IQR 3.10–8.15). The median age of the patients at the time of examination was 49.2 years (IQR: [42.33–56.98]). Radiomic and clinical–radiomic models showed significant associations between RS, DFS, and OS, with patients with RS below the median showing a better prognosis (*p* < 0.001). Bootstrap testing confirmed a good model fit for OS prediction, with median C-index values of 0.82 for the clinical model and 0.84 for the clinical–radiomic model. Conclusions: Radiomic analysis of CEM images may predict DFS and OS in BC patients, offering additional prognostic value beyond clinical models alone.

## Simple Summary

Breast cancer is a complex disease with different outcomes, making it essential to identify new ways to predict how each patient will respond to treatment. This study explores a new method that analyzes medical images—specifically, contrast-enhanced mammography—using advanced computer techniques called radiomics. By examining features that are not visible to the naked eye, the authors aim to develop a score that helps predict how long patients will live without disease and their overall survival chances. The results suggest that these radiomic features, when combined with clinical data, may improve the ability to predict outcomes. This approach could help doctors make better decisions early on, using a quick, non-invasive, and cost-effective tool that could be integrated into routine clinical care.

## 1. Introduction

Precision medicine and personalized care are essential in managing Breast Cancer (BC) patients, as they provide personalized treatment based on the patient’s genetic, histopathological, molecular, and clinical characteristics [1]. BC in women presents differently and has varying outcomes; hence, there is a need to develop novel biomarkers that can aid in the early and accurate prediction of prognosis, which, in turn, can help deliver precise and individualized treatment [2,3]. Studies have shown that the biological markers of the primary tumor are related to the patient’s prognosis [4]. For example, the St. Gallen classification of four BC subtypes (luminal A, luminal B, HER2-positive, and triple-negative) [5], which was proposed based on studies of gene expression analysis, is a good predictor of treatment response [6]. However, some issues are related to the assessment of these biomarkers for outcome prediction, including issues related to cost, non-repeatability, and ease of access [7]. In addition, due to the spatial and temporal heterogeneity of BC, conventional invasive biopsy techniques cannot extensively sample the entire tumor (sample bias) [8]. There is great potential for imaging in the prediction of disease outcomes [9], as it provides a more holistic view of the entire tumor and can be used serially during treatment to assess disease progression or the effect of treatment [9,10]. Radiomics is an emerging technique that automatically extracts a broad range of features from imaging data, enabling subsequent analysis and decision-making support [10]. The application of radiomics in oncology has evolved rapidly in recent years and has found its place in personalized medicine, particularly in tumor detection, subtype classification, and treatment response assessment [9,10]. Some recent studies have also investigated the possibility of using radiomics models based on breast MRI images to predict DFS and OS [11,12,13,14,15]. These initial studies suggest that adding radiomics to clinical models may improve the prediction of DFS and OS. In this context, CEM is being used more frequently. It has similar diagnostic performance to MRI, lower economic costs, and better patient tolerance due to shorter execution times and improved availability [16,17]. There are few studies related to radiomics with CEM [18,19]. In particular, we are unaware of any article submitted for publication that describes the development of a radiomics model based on CEM images to predict DFS and OS. The main objective of this work is to assess the predictive value of radiomics models developed from CEM imaging for DFS and OS in BC patients and compare it with clinical and molecular models.

## 2. Materials and Methods

The local ethics committee approved this retrospective study and waived the requirement for specific informed consent (Protocol Number UID 4625). All consecutive patients who underwent CEM between 2013 and 2015 before a breast biopsy that confirmed a primary BC diagnosis were enrolled. Patients with available follow-up were selected. The study population consists of asymptomatic patients who underwent screening tests (mammography and ultrasound) and were found to have a suspicious lesion (BIRADS > 3) [20] for which a biopsy assessment was requested. Following the detection of a suspicious lesion, CEM was performed before cytological or histological evaluation. None of the patients had a confirmed genetic mutation.

Inclusion criteria included the following:

- Patients with suspicious lesions detected in screening examinations who underwent CEM before cytological/histological assessment;
- Patients whose cytological/histological evaluation resulted in a diagnosis of breast cancer;
- Patients with a single breast cancer lesion;
- Patients who underwent breast surgery at the same institution.

Exclusion criteria included the following:

- Patients whose biopsy result, performed after CEM, indicated a benign condition;
- Patients with more than one breast lesion;
- Patients for whom follow-up data could not be retrieved;
- Pregnant patients;
- Patients with known allergies to iodinated contrast media.

### 2.1. Collected Data

Clinical data included patient age at biopsy, type of biopsied lesion, breast density [20], background parenchymal enhancement on CEM, size of the enhancement, and conspicuity of the biopsied lesion [21]. Biopsy data were recorded for histotype, histological tumor grade (G1, low grade; G2, intermediate grade; G3, high grade), receptor profile expression, and the proliferation index (Ki-67 antigen expression) through an immunohistochemical analysis conducted in our pathology department on the surgical specimen. Data regarding patient follow-up were collected. The event of death for the OS analysis was considered concerning causes associated with the original breast cancer pathology. The following oncological outcomes were considered for analysis: OS, defined as the time from image acquisition to death due to breast cancer-related causes; DFS, defined as the time from image acquisition to the event: local recurrence, second ipsilateral BC, contralateral BC, BC metastasis, or another tumor (whichever occurred first). Local recurrences were diagnosed through mammographic control and/or associated ultrasound (depending on breast density) performed once a year in this category of patients. Patients who died from causes other than breast cancer were censored at the date of death, while those lost to follow-up were censored at the date of their last available follow-up.

### 2.2. CEM Protocol

All CEM examinations were conducted utilizing a full-field digital mammography system (Senographe™ essential mammography system, GE Healthcare, Chalfont St. Giles, United Kingdom). This system was customized to enable dual-energy exposures and equipped with specialized image acquisition and processing software. Before breast compression, patients received a single automated intravenous injection of an iodinated contrast agent (Iohexol, 300 mg/mL, 1.5 mL/kg, Omnipaque^®^, GE Healthcare). Image acquisition started two minutes post injection, capturing a series of bilateral cranio-caudal (CC) and medio-lateral-oblique (MLO) views initiated from the suspicious breast. The entire examination was completed within seven minutes of administering the contrast agent.

### 2.3. Image Analysis

Two certified breast radiologists (A.B., with over 20 years of experience, and L.N., with over 8 years of experience) conducted a retrospective re-evaluation of the CEM images of the included patients. The same radiologists outlined the boundaries of enhancement for each lesion, known as the region of interest (ROI), encompassing the entire enhancing lesion, in agreement with each other. In cases of disagreement on the lesion margins to be delineated, a third radiologist (E.C., with over 20 years of experience) was consulted to reach a consensus.

Disagreements between the two primary radiologists on ROI delineation occurred in approximately 8% of cases (10 out of 126 patients). In these instances, the third senior radiologist was consulted, and consensus was achieved through joint review.

**Figure 1:**
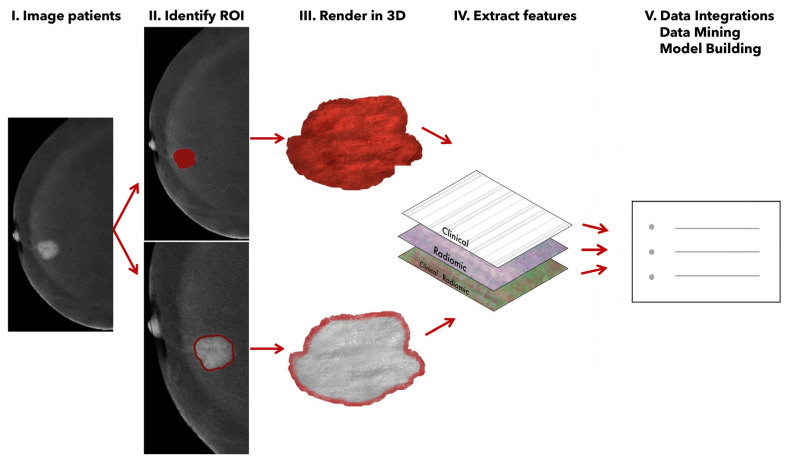
Radiomic feature extraction from recombined CEM images.

The segmentation was performed in the projection where the enhancement was most visible, always achieving agreement between the two radiologists responsible for reviewing the images (Figure 1). Subsequently, all images were exported to the open-source LIFEx v 6.32 image processing tool [22], where features were extracted using a fixed number of bins (64 bins) and spatial resampling to accommodate various fields of view. An example of the feature extraction workflow is schematized in Figure 1.

### 2.4. Feature Selection and Radiomic Score Calculation

Radiomic features with nearly zero variance and high correlation (Spearman ρ > 0.95) were excluded, similarly to Zhan, Y et al. [23] and Yue, X et al. [24]. To address multicollinearity and dimensionality, an iterative clustering process (ρ > 0.75) was used to retain only the most outcome-associated feature from each cluster based on univariate Cox regression (lowest *p*-value for OS and DFS). The process was repeated until all the between-feature correlations (ρ) were ≤0.75. The multivariable Cox regression model with the least absolute shrinkage and selection operator (LASSO) was used to produce a coefficient for each feature, where a higher coefficient value indicates a greater contribution to the endpoint prediction. To ensure robustness, we implemented 100 repeated runs of leave-one-out cross-validation. At each iteration, we fitted a Cox model with LASSO regularization using the glmnet package in R. The optimal penalty parameter (λ) was selected as the min λ value (minimizing the partial likelihood deviance in each run). The distribution of the 100 selected λ values was used to assess the stability of model regularization. This yielded a sparse model with selected features most strongly associated with OS and DFS outcomes.

### 2.5. Prognostic Models Comparison

Univariate Cox proportional hazards regression models were used to test associations of clinical variables with OS and DFS. Cut-offs for continuous variables were chosen according to clinical significance or, in its absence, using the median value. Variables with a *p*-value of less than 0.10 in univariate analysis were included in multivariable analysis. Risk estimates were quantified by hazard ratios (HRs) and 95% confidence intervals (CIs). Survival was presented with Kaplan–Meier curves. A log-rank test was used to compare survival curves by stratification.

Three predictive models for each survival endpoint were evaluated: the clinical model (containing only clinical information: age, the diameter of the recombined image enhancement, and the receptor profile of the tumor lesion), the radiomic model (containing only the RS), and the clinical–radiomic model (containing both clinical data and the RS). For graphical representation and univariate analysis, the RS was dichotomized into high and low RS using the median. Finally, the model fit was evaluated using Harrell’s C index, a generalization of the area under the ROC curve (AUC) that can take into account censored data [25]. *p*-values < 0.05 were considered to indicate statistical significance for each model. To assess model performance and robustness, we implemented a repeated, stratified 3-fold in-sample cross-validation. The process was repeated 500 times, each time using a different random seed to shuffle the dataset. In each repetition, patients with and without the event were separately stratified to maintain event balance across the folds. For each fold within a repetition, two-thirds of the data were used for model training and one-third for testing. For each of the three models, we calculated Harrell’s concordance index (C index) on both the training and testing subsets. In each repetition, the median C index across the three folds was computed and stored. This resulted in a distribution of 500 median C indices per model, separately for training and testing sets. Finally, we reported the empirical quantiles (e.g., 25th, 50th, and 75th percentiles) of these C-index distributions to summarize model performance. We also assessed the predictive performance of the variables in estimating the occurrence of the events as a binary outcome rather than as time-to-event data. The area under the curve (AUC) and its corresponding 95% (de Long) confidence interval were reported. All analyses were performed using R (version 4.1.1).

## 3. Results

**Figure 2:**
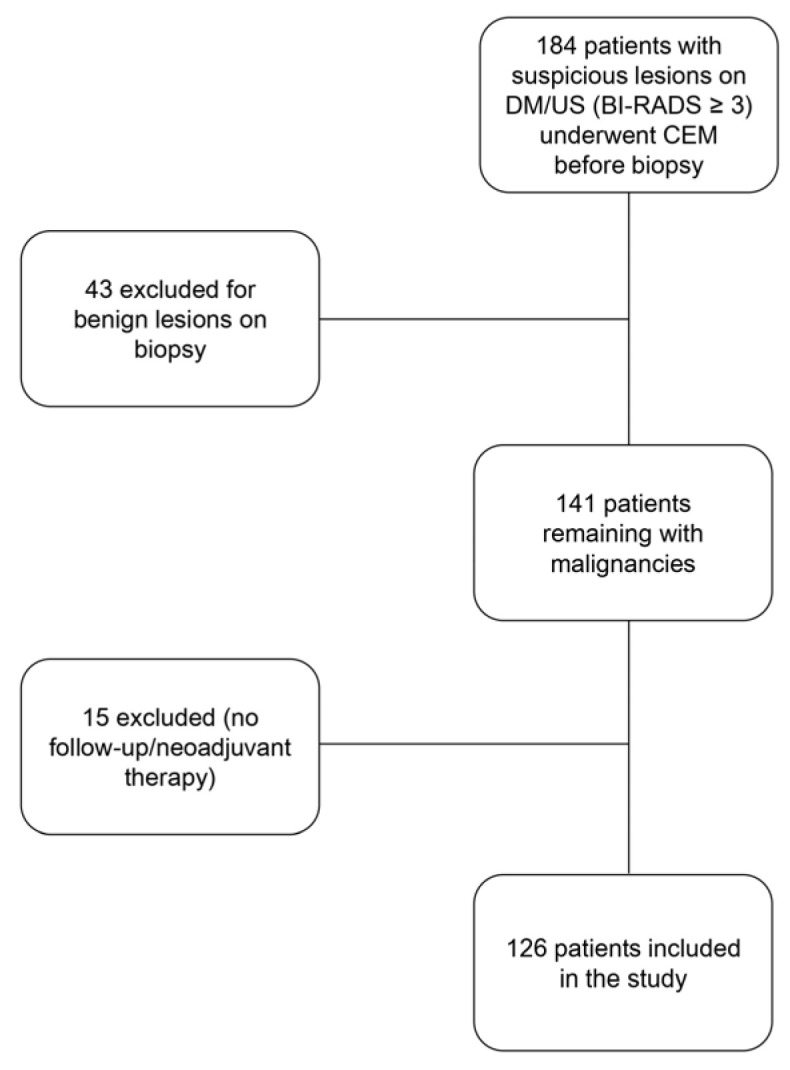
Flowchart of the study.

A total of 184 patients were selected to undergo CEM following the detection of a suspicious breast finding that required cytological/histological assessment. Subsequently, 58 patients were excluded (either because the histological result of the evaluation was benign, because they underwent neoadjuvant chemotherapy, or because follow-up data could not be retrieved). The study flowchart is shown in Figure 2.

The median age of the patients at the time of examination was 49.2 years (IQR: 42.33–56.98). Most lesions were of the “mass” type (120/126, 95%) and were identified in patients with heterogeneously dense breasts (ACR C, 84/126, 67%) and minimal background enhancement (78/126, 62%). Lesion conspicuity was high in 46% (58/126) of cases and moderate in 40%. The median size of lesion enhancement was 18 mm. Of the 126 lesions, 125 (99%) were invasive at surgery, and only 1.6% of patients had an in situ lesion.

At surgery, most lesions were of intermediate grade G2 (45%, 57/126) or high grade G3 (40%, 50/126). Regarding molecular expression profiles, luminal A tumors predominated (44%, 55/126), followed by luminal B (34%, 43/126), HER2-positive (13%, 16/126), and triple-negative tumors (9.5%, 12/126).

The descriptive data of the 126 patients and their lesions are summarized in Table 1, Table 2 and Table 3.

The median survival follow-up was 6.88 years (IQR: 3.10–8.15). Eleven patients died, with a median survival time from the start of observation of 3.44 years (1.79–5.17). Twenty-three patients experienced disease recurrence; their characteristics are detailed in Table 4. Most cases involved BC metastases (15/23, 65%) and breast disease recurrence (8/23, 35%).

### 3.1. Radiomic Analysis

The coefficients of RS obtained from the LASSO-Cox proportional model were as follows: OS: CONVENTIONAL_mean: -0.0016415, NGLDM_Coarseness: −2140.1761; DFS: NGLDM_Coarseness: −800.56091, NGLDM_Contrast: −4.7468934, GLZLM_SZHGE: −6.55 × 10^−5^, GLZLM_ZLNU: 1.48 × 10^−5^.

### 3.2. Overall Survival (OS)

In the clinical model, enhancement size and molecular subtype were included. An increase in enhancement size was significantly associated with worse survival (HR 1.04, 95% CI 1.01–1.06; *p* = 0.005). Compared to the triple-negative reference group, a borderline lower risk of death was observed for HER2-positive tumors (HR 0.10, 95% CI 0.01–1.14; *p* = 0.064) and luminal B tumors (HR 0.24, 95% CI 0.05–1.12; *p* = 0.069).

**Figure 3:**
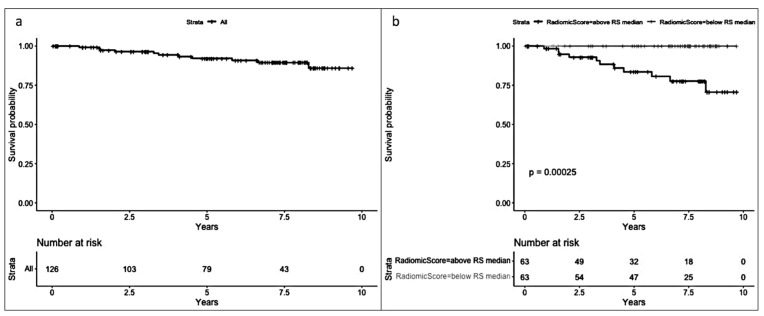
(**a**) Overall survival (OS) and (**b**) OS by median radiomic score (equal to or below and above −4.25).

In the radiomic model (Figure 3), a significant difference in survival was observed between patients with an RS below the median and those with an RS above the median (Log-rank test *p* < 0.001), favoring a better prognosis for patients with an RS below the median (−4.25). Median follow-up for patients above the median RS was 5.82 years [2.84, 8.11], while median follow-up for those below the median RS was 7.12 years [4.97, 8.16].

In the clinical–radiomic model (Supplementary Table S1), the statistical significance of enhancement size in the prediction of OS was lost in favor of the RS, with an increase in the RS being significantly associated with an increased risk of death (*p* = 0.007).

**Figure 4:**
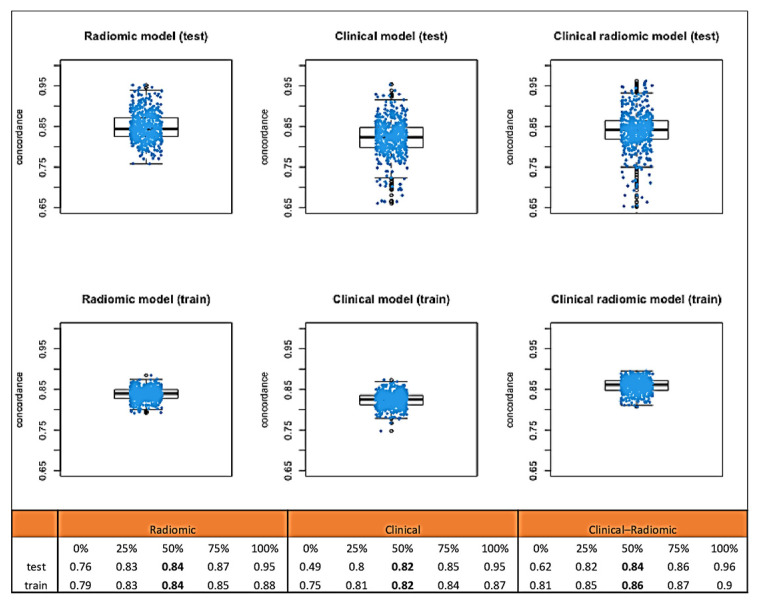
Median C-index values obtained through bootstrap testing for OS.

Median C-index values obtained through bootstrap testing demonstrated a good fit of the model to the data (Figure 4): the clinical model had a median test C index of 0.82 (IQR: 0.80–0.85), while the test C index of the clinical–radiomic model was slightly higher, at 0.84 (IQR 0.82–0.86).

Considering the endpoint as dichotomic, here, we report the performance results of the logistic models. The clinical mode showed an AUC of 0.76 (95% CI: 0.60–0.93), the clinical–radiomic model showed an AUC of 0.86 (95% CI: 0.76–0.96), and the radiomic-only model showed an AUC of 0.85 (95% CI: 0.76–0.94). These findings indicate that the inclusion of radiomic features enhances the ability to predict the event, with the clinical–radiomic model showing the highest discriminative performance.

### 3.3. Disease-Free Survival (DFS)

The median follow-up for DFS was 6.95 years (IQR 3.03–8.23). The median follow-up for patients with RS values above the median was 6.98 years (IQR: 2.55–8.23), while for those with an RS below the median, it was 6.88 years (IQR: 3.72–8.15).

In the clinical model, only the enhancement size was statistically significant, with an increase in enhancement size significantly associated with worse DFS (HR: 1.03; 95% CI: 1.01–1.04; *p* < 0.001).

**Figure 5:**
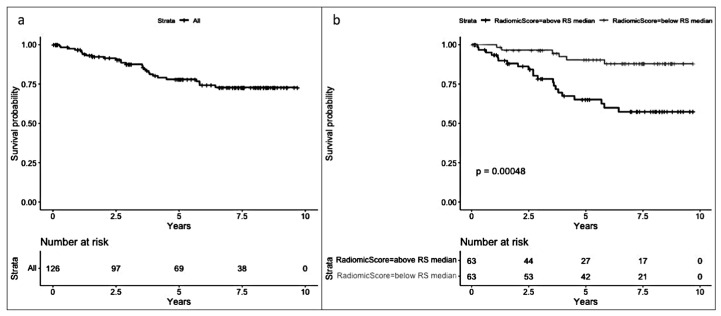
(**a**) Disease-free survival (DFS) and (**b**) DFS by median radiomic score (equal to or below and above −0.91).

In the radiomic model (Figure 5), a significant difference (*p* < 0.001) was observed between patients with an RS below the median and those with an RS above the median (median RS = −0.91), favoring a better prognosis for patients with an RS below the median.

In the clinical–radiomic (Supplementary Table S2) model, the significance of enhancement size was lost in favor of the RS (*p*-value = 0.007). An increase in the RS was significantly associated with an increased risk of recurrence or death.

**Figure 6:**
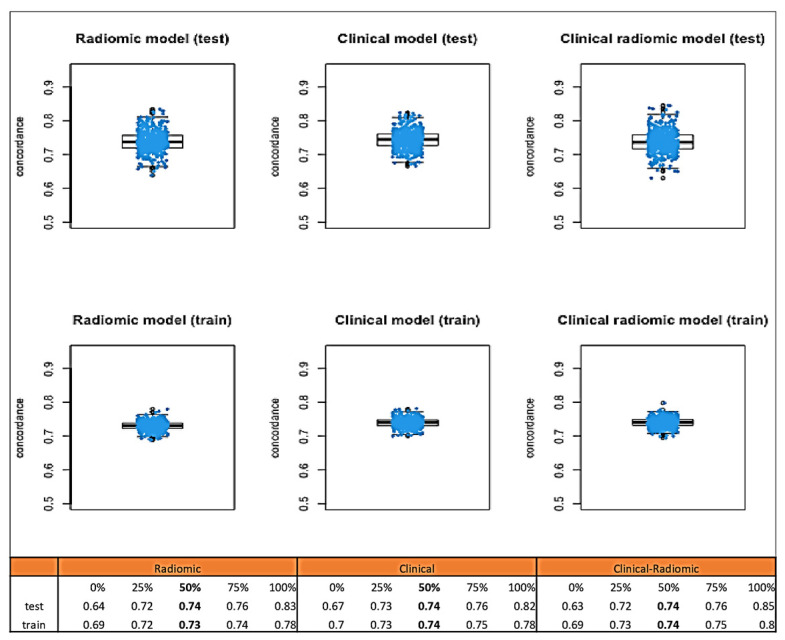
Median C-index values obtained through bootstrap testing for DFS.

Median C-index values obtained through bootstrap testing (Figure 6) demonstrated a good fit of the model to the data: the clinical model had a median C index of 0.74 (IQR: 0.73–0.75). The C index of the clinical–radiomic model was 0.74 (IQR: 0.73–0.75).

Considering the endpoint as dichotomic, here, we report the performance results of the logistic models. The clinical model showed an AUC of 0.75 (95% CI: 0.66–0.84). The Clinical–radiomic model had an AUC of 0.74 (95% CI: 0.63–0.84), while the radiomic-only model’s AUC was 0.74 (95% CI: 0.63–0.84). These results indicate that the clinical variable alone provided slightly better discriminative ability than either the radiomic-only or the combined clinical–radiomic model in predicting the binary disease-free status.

## 4. Discussion

This study found that radiomic analysis, applied to contrast-enhanced mammography, is a valuable tool for predicting breast cancer prognosis [26,27,28]. Our results show that the radiomic score (RS) improves the prediction of overall survival (OS), with the C index increasing from 0.82 (clinical model) to 0.84 (clinical–radiomic model). Also, the RS value was found to play a significant role in the estimation of DFS, with results comparable to those of traditional clinical parameters, such as receptor status [5]. This means that a radiomic model could provide early prognostic scores and could be a faster and more economical way of doing so than other methods, such as receptor status analysis [29,30]: radiomics can improve the prediction of survival beyond that of conventional clinical models [13,14,15]. Integrating radiomics into clinical practice, specifically concerning molecular biomarkers, presents an interesting challenge: breast cancer is highly prevalent in the general population [28], and the availability of fast, cost-effective, and non-invasive methods to stratify patients based on their risk of recurrence at follow-up could enable a more efficient allocation of medical resources. Radiological features can be useful indicators for predicting the therapeutic response in breast cancer patients and, thus, help devise better treatment plans [13,14,15,29]. This study is noteworthy because it combines radiomic features from contrast-enhanced mammography (CEM) images with clinical data and long-term follow-up. Our research has the advantage of using CEM data from 2013–2015, which provides a significant observation period. The possibility of having a CEM population from that time with follow-up data is valuable for the clinical information we can derive from the follow-up data, considering the scarcity of CEM exams performed during that time frame (2013–2015): CEM has only recently become widely used in clinical practice, despite being approved by the FDA in 2011.

These findings may help distinguish patients with poor prognoses, allowing for a more tailored treatment strategy. Our study presents results similar to those of studies conducted on breast MRI data from patients with breast neoplasia. For example, in the study by Mazurowski et al., it was shown how breast cancer features extracted from a breast MRI might be used for the assessment of the risk of distant recurrence [29]: radiomics can be applied in a longitudinal setting, enabling physicians to modify treatment plans according to changes in disease status [10,30,31]. To date, CEM has demonstrated high diagnostic value in managing breast lesions and often provides results similar to those of MRI [32]. The current recommended clinical indications for CEM include the locoregional staging of newly diagnosed breast cancer, the assessment of response to neoadjuvant treatment, the screening of high-risk patients with dense breasts, and the monitoring of patients with a history of breast cancer [33,34,35,36,37]. Several similarities can be observed between CEM and contrast-enhanced MRI: the two techniques are used for similar indications [16]. Nevertheless, CEM is faster and less expensive, but it has the disadvantage of using ionizing radiation [16].

Despite the availability of CEM, the application of radiomics in CEM studies remains scarce, with most existing research focusing on the non-invasive prediction of malignancy or disease aggressiveness [18,38]. This study is the first to compare radiomic features derived from CEM with prognostic information obtained from long-term follow-up data.

A deeper biological interpretation of the link between radiomic features and breast cancer prognosis is essential to understand the value of this approach [39]. Radiomic characteristics extracted from CEM such as texture, coarseness, and heterogeneity, may reflect underlying tumor microenvironmental factors such as cellular density, angiogenesis, and extracellular matrix remodeling [40]. For example, increased coarseness in texture analysis could be associated with irregular vascularization patterns or hypoxic areas, which are known to influence tumor aggressiveness and metastatic potential [41]. Additionally, heterogeneity metrics may capture intratumoral variation in cellular proliferation or necrosis, providing indirect information on tumor biology that is otherwise unavailable through routine imaging assessment. These biological principles highlight why certain radiomic signatures are prognostic: they may act as non-invasive biomarkers capturing the complexity of tumor behavior, complementing molecular data and offering new avenues for personalized patient management [41]. Future studies combining radiomics with tissue-based biomarkers (e.g., gene expression profiles) could help clarify these mechanisms and strengthen the biological rationale behind radiomics-guided prognostic models.

This study has several limitations that need to be discussed. The primary limitation is its retrospective design, which may introduce selection bias; however, the inclusion of over one hundred patients provides an initial basis for analysis and helps mitigate this risk. Moreover, the retrospective nature was the only approach that could be adopted for the study design and to obtain follow-up data from patients who underwent this type of examination (CEM). Other limitations include the use of manual segmentation, which is time-consuming and could be prone to interoperator variability [42]: to minimize this issue, two operators conducted the study to reduce variability, and in cases of disagreement, a third operator was consulted to reach a consensus. Some researchers are beginning to incorporate automated or semi-automated segmentation techniques to minimize variability [42].

However, it is interesting to consider how some recently published investigations show that the experience of an expert radiologist could be superior to other automated segmentation methods [43,44,45]. It should be noted that another significant limitation of the study is the lack of external validation of the model. To mitigate this limitation, we employed robust statistical methods, including univariate and multivariate Cox proportional hazards regression models and the Least Absolute Shrinkage and Selection Operator (LASSO) for feature selection, ensuring the most reliable and rigorous analysis of radiomic data. Finally, another important limitation of our study is the relatively small sample size, which may limit the generalizability of the findings. Future studies with larger, multicenter cohorts are needed to validate and expand upon these preliminary results.

Nevertheless, considering this limitation (lack of external validation), this paper should be regarded as the presentation of auspicious preliminary data on the application of radiomics to CEM for the assessment of the prognosis of patients with breast cancer, an approach that has already been utilized in recently published studies.

Other works with external validation of the model that aim to confirm our preliminary data will undoubtedly need to be conducted to validate our initial results.

## 5. Conclusions

Our study demonstrates the effectiveness of applying radiomic analysis of CEM images for personalized breast cancer treatment. It identifies patients with poor prognoses early on in a non-invasive and cost-efficient manner. The extracted features are correlated with OS and DFS and significantly enhance predictive ability. If we can identify high-risk patients without invasive procedures, we could change cancer surveillance and improve survival rates for those at the highest risk of recurrence. If adopted in clinical practice, these advanced techniques can potentially improve decision-making and patient care.

## Figures and Tables

**Table 1:** Descriptive characteristics of the patient population included in the study.

Characteristics	N = 126 ^1^
Lesion type	
Mass	120 (95%)
Mass with microcalcifications	2 (1.6%)
Microcalcifications	4 (3.2%)
Breast density (ACR)	
A	1 (0.8%)
B	26 (21%)
C	84 (67%)
D	15 (12%)
Background (minimal, mild, moderate, or marked)	
Minimal	78 (62%)
Mild	32 (25%)
Moderate	9 (7.1%)
Marked	7 (5.6%)
Enhancement	
Yes	126 (100%)
Lesion Conspicuity (mild, moderate, or marked)	
Mild	17 (13%)
Moderate	51 (40%)
Marked	58 (46%)
Enhancement size	18 (14, 32)

Abbreviations: ER = estrogen receptor; PgR = progesterone receptor; HER2 = human epidermal growth factor receptor 2; Ki-67 = proliferation index.

**Table 2:** Staging of the breast lesions.

Characteristics	N = 126 ^1^
T stage	
pT1	72 (57%)
pT2	37 (29%)
pT3	13 (10%)
pT4	1 (0.8%)
yT1	3 (2.4%)
N stage	
pN0	59 (47%)
pN1	35 (28%)
pN2	11 (8.7%)
pN3	16 (13%)
pNX	5 (4.0%)
Stage	
I	52 (41%)
II	44 (35%)
III	30 (24%)
Grading at biopsy	
G1	16 (13%)
G2	58 (46%)
G3	48 (38%)
Missing	4 (3.2%)
^1^ n (%); Range; Median (IQR)	

Abbreviations: ER = estrogen receptor; PgR = progesterone receptor; HER2 = human epidermal growth factor receptor 2; Ki-67 = proliferation index.

**Table 3:** Molecular profiles of the breast lesions.

Characteristics	N = 126 ^1^
**ER median (Q2, Q4)**	90 (70, 95)
**PgR median (Q2, Q4)**	70 (15, 90)
**Ki-67 median (Q2, Q4)**	22 (14, 28)
HER2	
Absent	59 (47%)
Low	48 (38%)
High	19 (15%)
Molecular profile	
HER2	16 (13%)
Luminal A	55 (44%)
Luminal B	43 (34%)
TN	12 (9.5%)
Grading at surgery	
G1	17 (13%)
G2	57 (45%)
G3	50 (40%)
Missing	2 (1.6%)
^1^ n (%); Range; Median (IQR)	

Abbreviations: ER = estrogen receptor; PgR = progesterone receptor; HER2 = human epidermal growth factor receptor 2; Ki-67 = proliferation index.

**Table 4:** Primary characteristics of patients with recurrence.

Characteristics of Patients with Recurrence	N = 23/126
Event type	
Breast cancer metastasis	15
Breast recurrence	8
Histology of surgery at follow-up	
Infiltrative ductal carcinoma	6
Lobular intraepithelial neoplasia (LIN2)	1
High-grade ductal carcinoma in situ (DIN3)	1
N/A	15
Molecular profile of surgery at follow-up	
Er 0% Pgr 0%; Her 2+; Ki-67 18%	1
Er 90% Pgr 0%; Her 2+; Ki-67 27%	1
Er 90% Pgr 0%; Her 2 0; Ki-67 15%	1
Er 90% Pgr 0%; Her 2+; Ki-67 2%	1
Er 90% Pgr 0%; Her2 neg; Ki-67 22%	1
Er 95% Pgr 3%; Her 2neg; Ki-67 30%	1
Er 95% Pgr 90%; Her 2 neg; Ki-67 28%	1
Er 90% Pgr 0%; Her 2 neg; Ki-67 35%	1
N/A	15
Site of metastasis	
Ipsilateral axillary lymph node	1
Bone at onset	1
Ovary	1
Bone and liver	2
Lymph node and pleura (from gastric cancer)	1
Lymph node, liver, and bone	1
Lymph node, omentum, bowel, and liver	1
Brain	1
Brain, bone, liver, and lymph node	1
Lymph node	3
Pleura	1
Uterus	1
N/A	8

N/A: not applicable. Abbreviations: ER = estrogen receptor; PgR = progesterone receptor; HER2 = human epidermal growth factor receptor 2; Ki-67 = proliferation index.

## Data Availability

The data presented in this study are available upon request from the corresponding author. The data are not publicly available due to privacy concerns.

## Supplementary Materials

The following supporting information can be downloaded at: https://www.mdpi.com/article/10.3390/cancers17121926/s1, Table S1: Prognostic model comparison for Overall Survival (OS); Table S2: Prognostic model comparison for Disease-Free Survival (DFS).
