# Supplementary material for: Preliminary Evaluation of Radiomics in Contrast-Enhanced Mammography for Prognostic Prediction of Breast Cancer
Source: Cancers (Basel). 2025 Jun 10;17(12):1926. doi: 10.3390/cancers17121926 (PMC12190519; doi:10.3390/cancers17121926)
Supplement: Supplementary file 1 [file cancers-17-01926-s001.zip › cancers-3654219-supplementary.pdf]

***Supplementary Table S1.*** *Prognostic model comparison for Overall Survival (OS).*

| Overall Survival (OS) | | | | | | | | | | | | | | | |
|---|---|---|---|---|---|---|---|---|---|---|---|---|---|---|---|
| **Characteristic** | **Clinical Model OS** | | | | | **Radiomic Model OS** | | | | | **Clinical-Radiomic Model OS** | | | | |
| | **N** | **Event N** | **HR[1]** | **95% CI[1]** | **P-value** | **N** | **Event N** | **HR[1]** | **95% CI[1]** | **P-value** | **N** | **Event N** | **HR[1]** | **95% CI[1]** | **P-value** |
| **Enhancement size** | 126 | 11 | 1.04 | 1.01,1.06 | **0.005** | | | | | | 126 | 11 | 1.01 | 0.98,1.04 | 0.500 |
| **Molecular subtype** | | | | | | | | | | | | | | | |
| TN | 12 | 3 | — | — | | | | | | | 12 | 3 | — | — | |
| HER2 | 16 | 1 | 0.10 | 0.01,1.14 | 0.064 | | | | | | 16 | 1 | 0.12 | 0.01,1.23 | 0.074 |
| Luminal A | 55 | 3 | 0.26 | 0.05,1.42 | 0.121 | | | | | | 55 | 3 | 0.37 | 0.07,2.05 | 0.256 |
| Luminal B | 43 | 4 | 0.24 | 0.05 1.12 | 0.069 | | | | | | 43 | 4 | 0.35 | 0.08,1.61 | 0.178 |
| **Radiomic score os *100** | | | | | | 126 | 11 | 1.03 | 1.01,1.06 | **0.002** | 126 | 11 | 1.04 | 1.01,1.06 | **0.007** |

***Supplementary Table S2.*** *Prognostic model comparison for Disease-Free Survival (DFS).*

| Disease-Free Survival (DFS) | | | | | | | | | | | | | | | |
|---|---|---|---|---|---|---|---|---|---|---|---|---|---|---|---|
| | **Clinical Model DFS** | | | | | **Radiomic Model DFS** | | | | | **Clinical-Radiomic Model DFS** | | | | |
| **Characteristic** | **N** | **Event N** | **HR[1]** | **95% CI[1]** | **P-value** | **N** | **Event N** | **HR[1]** | **95% CI[1]** | **P-value** | **N** | **Event N** | **HR[1]** | **95% CI[1]** | **P-value** |
| **Enhancement size** | 126 | 27 | 1.03 | 1.01, 1.04 | **0.0003** | | | | | | 126 | 27 | 1.01 | 0.99, 1.03 | 0.225 |
| **Radiomic Score** | | | | | | 126 | 27 | 7.47 | 2.59, 21.5 | **0.0001** | 126 | 27 | 5.17 | 1.58, 16.9 | **0.007** |
